# New records of Niceforo’s big-eared bat, *Trinycterisnicefori* (Sanborn, 1949) (Chiroptera, Phyllostomidae), from the state of Maranhão, Brazil

**DOI:** 10.3897/zookeys.787.26538

**Published:** 2018-10-02

**Authors:** Amanda Cristiny da Silva Lima, Fabio Henrique Souza Cardoso, Samira Brito Mendes, Elmary Costa Fraga, Maria Claudene Barros

**Affiliations:** 1 Undergraduate in Biological Sciences, Centro de Estudos Superiores de Caxias (CESC), Universidade Estadual do Maranhão (UEMA), Praça Duque de Caxias S / N, Bairro Alecrim, 65604-000, Caxias / MA, Brazil Universidade Estadual do Maranhão Caxias Brazil; 2 Graduate Program in Animal Science, Centro de Ciências Agrárias (CCA), Universidade Estadual do Maranhão (UEMA), Cidade Universitária Paulo VI, Av. Lourenço Vieira da Silva, nº 1000 – Bairro Tirirical, 65055-150, São Luís / MA, Brazil Universidade Estadual do Maranhão Caxias Brazil; 3 Genetics and Molecular Biology Laboratory, Centro de Estudos Superiores de Caxias (CESC), Universidade Estadual do Maranhão (UEMA), Praça Duque de Caxias S / N, Bairro Alecrim, 65604-000, Caxias / MA, Brazil Universidade Estadual do Maranhão Caxias Brazil

**Keywords:** Bats, COI gene, Maranhão, mitochondrial, range extension

## Abstract

Niceforo’s big-eared bat, *Trinycterisnicefori* (Sanborn, 1949), is a monotypic species which has been recorded in a number of Brazilian states, but has a disjunct distribution in this country. This study presents the first record of *T.nicefori* in the Brazilian state of Maranhão. The specimens were collected in the municipalities of Godofredo Viana and Cândido Mendes, in fragments of the Amazon forest. One male (forearm: 38.00 mm, weight: 6 g) and one female (39.68 mm, 8 g) specimens were collected. The specimens presented chestnut-colored fur, and a chin with a pair of dermal pads arranged in a V-shape, without a central papilla. The COI gene sequences were plotted in the BOLD Systems platform, which confirmed the morphological identification of the species, with a 99.1% similarity in the male, and 99.4% in the female to existing sequences. This record extends the known distribution of *T.nicefori* in Brazil by approximately 310 km to the most eastern part of the Amazon Biome.

## Introduction

The genus *Trinycteris* Sanborn, 1949 was originally described as a subgenus of *Micronycteris* Gray, 1866, and subsequently recognized as a monotypic genus by Simmons (1996) and Simmons and Voss (1998). Wetterer et al. (2000) confirmed the validity of the genus through a combined analysis of morphological and molecular features. Nogueira et al. (2014) and Reis et al. (2017) re-allocated *Trinycterisnicefori* to the subfamily Glyphonycterinae (rather than Phyllostominae), following the proposal of Baker et al. (2003). Given the problems of the classification of this genus, further analyses are required to conclusively determine its taxonomic placement (Reis et al. 2017).

Niceforo’s big-eared bat, *Trinycterisnicefori*, is an insectivore (Reis et al. 2013, 2017). The diagnostic characteristics of the species include: its small size, with an adult head-body length of 51–58 mm; forearm of 35–41 mm; faintly tricolored dorsal hairs, with a darker base and tip; ventral fur dark; ventral margin of the nasal leaf horseshoe merging gradually with the upper lip, chin with a pair of dermal pads arranged in a V shape, without a central papilla; face and anterior orbital region of the cranium not inflated (Reis et al. 2013, 2017).

The known geographic distribution of *T.nicefori* ranges from southern Mexico to Central America, Trinidad, Colombia, Venezuela, Guiana, Suriname, Peru, Ecuador, Bolivia, and Brazil (Peracchi et al. 2011, Rocha et al. 2013). In Brazil, the species has been recorded in the states of Acre, Amazonas, Amapá, Bahia, Espirito Santo, Mato Grosso, Pará, Roraima, Santa Catarina, Rondônia and Tocantins (Tavares et al. 2008, Peracchi et al. 2011, Rocha et al. 2013). Although there are records of *T.nicefori* in transitional areas between the Amazon forest and the Cerrado savanna of central Brazil (Nunes et al. 2005, Peracchi et al. 2011), there are considerable areas not yet surveyed from the Amazon and Atlantic forests (Rocha et al. 2013).

More than 100 bat species are known to occur in the Amazon region. In the Amazonian domain of the Brazilian state of Maranhão, 47 species have been recorded representing 29 genera (Oliveira et al. 2011, Lópes-Baucells et al. 2016). Despite considerable sampling effort in Maranhão, in comparison with other states, its bat species list is still considered incomplete (Oliveira et al. 2011). Maranhão has a diversity of landscapes, and is considered to be an ecotone between three major biomes, the Amazon, Cerrado, and Caatinga (Batistella et al. 2014, Spinelli-Araujo et al. 2016). The taxonomy and geographic distribution of the state’s small mammals, in particular its bats, are still relatively poorly known (Oliveira et al. 2011, Olímpio et al. 2015). An increase in the sampling of these areas will provide a better understanding of the current distribution of bat species, including *T.nicefori* (Rocha et al. 2013). The present study reports the species *T.nicefori* for the first time in the state of Maranhão, extending its distribution to the eastern end of the Amazon biome through morphological and molecular identification.

## Material and methods

The specimens of *T.nicefori* were collected from fragments of forest in the Amazonian domain of Maranhão in April 2017, in the municipalities of Godofredo Viana and Cândido Mendes. The bats were captured in mist-nets, 3 m high, and 9–12 m in length, with a 25 mm mesh, which were fixed to the ground with poles and cords. The age of the specimens was estimated based on the ossification of the phalangeal epiphyses, and the reproductive condition was determined by palpation of the teats and abdomen in the female and the position of the testicles in the male (Brunet and Austad 2004). The specimens were photographed, euthanized, labelled, weighed, and measured. Specimens were kept cold until their arrival at the Laboratory of Genetics and Molecular Biology (GENBIMOL) at CESC/UEMA in Caxias, Maranhão, where samples of muscle tissue were extracted and stored in 70% ethanol for subsequent molecular analyses. The cranial structure was also analyzed after preparation.

The morphological and craniometric measurements were obtained using a manual caliper, following Vizzoto and Taddei (1973), and Simmons and Voss (1998) (Table 2): right and left forearm, ear, tragus, foot, tail, skull length, basal skull length, width of the mastoid and zygomatic processes, braincase breadth, palate length, length of the upper and lower tooth-row series, and the length of the mandible. The specimens were fixed in 10% formalin and conserved in 90% ethanol. The species was identified using the classification keys of Uieda et al. (2006), Peracchi et al. (2011), and Reis et al. (2013). The collection of samples was authorized by IBAMA/SISBIO license 42670-3. The specimens will be deposited in the Mastozoology collection of the Federal University of Paraíba, in João Pessoa, Brazil.

For the molecular analyses, total DNA was extracted from the samples of muscle tissue using the Wizard Genomic DNA Purification kit (Promega), according to the manufacturer’s instructions. The mitochondrial Cytochrome Oxidase Subunit I (COI) gene was amplified by Polymerase Chain Reaction (PCR) using the primers LCO-1490 and HCO-2198 described by Folmer et al. (1994). The samples were sequenced using Sanger et al.’s (1977) dideoxyterminal method, run in an ABI Prism 3500 automatic DNA sequencer (Applied Biosystems, USA) with the Big Dye kit. The sequences were edited and aligned in BIOEDIT 7.0 (Hall 1999), and plotted in the BOLD Systems v4 platform (http://www.boldsystems.org) to evaluate their similarity with existing sequences.

## Results

**Figure 1.**
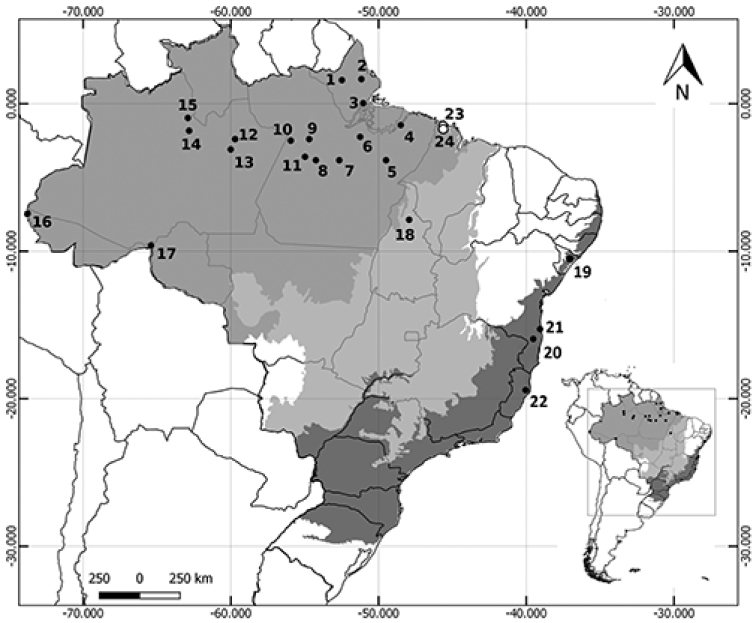
Geographic distribution of *Trinycterisnicefori* in Brazil. The area in which the specimens analyzed in the present study were collected in Brazil is indicated by open circles (○). The Brazilian biomes are shaded dark gray (Atlantic Forest), light gray (Cerrado), and medium gray (Amazon).

**Figure 2.**
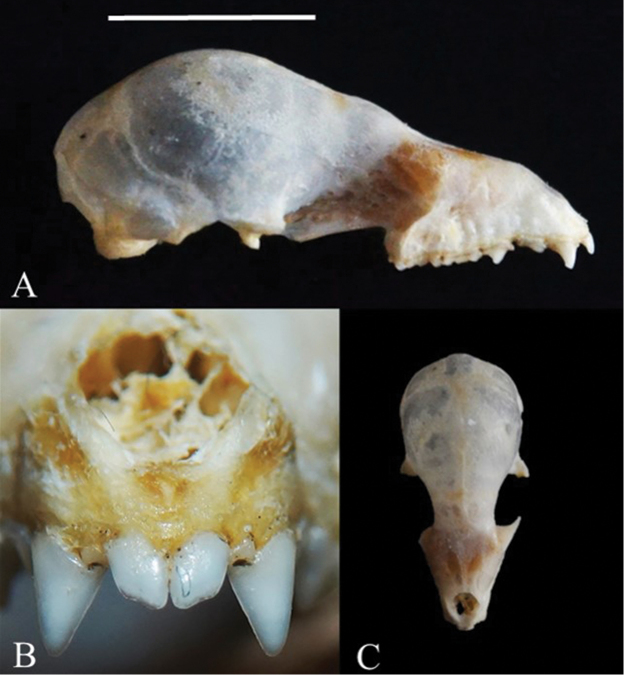
Cranium of *Trinycterisnicefori*: (**A**) lateral view showing protruding upper incisors not aligned with the canines (**B**) protruding upper incisors (**C**) frontal view of the cranium showing rostrum shorter than the braincase and anterior orbital region. Scale bar: 10 mm.

Two specimens of *T.nicefori* were captured at the study sites in the Amazonian domain of Maranhão (Table 1, Figure 1). The female specimen (field number: RRM 07, forearm: 39.68 mm, body mass: 8 g) was larger than the male specimen (field number: RRM 126, 38.00 mm, 6 g). The two specimens were sexually mature adults, although the female was not lactating; the testicles of the male were scrotal, an indicator that it was sexually active (Kunz et al 1983). The pelage color was chestnut, with a more lightly-colored venter and faintly tricolored dorsal hairs. The specimens were also identified based on a set of diagnostic cranial characters: two pairs of upper incisors and three lower premolars; the incisors were not chisel-shaped, were protruding and were not aligned with the canines, being much shorter and narrower, rostrum shorter than the braincase, rostrum and anterior orbital region of the cranium not inflated (Table 2, Figure 2).

The analysis of the COI molecular marker on the Bold System platform confirmed the morphological identification of the specimens collected in the present work. These specimens present 99% of similarity with the sequences of *T.nicefori* of Costa Rica and a divergence of 1–3% in comparison with sequences of *T.nicefori* from Guiana.

**Table 1.** Geographical location of specimens of *Trinycterisnicefori* collected in the state of Maranhão, with voucher and GenBank accession numbers.

Species	Biome	Collecting locality	Geographic coordinates	Field number/Voucher	GenBank accession number
* T. nicefori *	Amazonian domain of Maranhão, Brazil	Godofredo Viana	1°24.891'S, 45°46.446'W	RRM 07	MH807256
		Cândido Mendes	1°26.971'S, 45°44.201'W	RRM 126	MH807257

**Table 2.** Craniometric measurements, in mm, of *Trinycterisnicefori* obtained in the present study, compared with the holotype (Sanborn 1949), and the specimens analyzed by Simmons and Voss (1998), showing the range of values (minimum-maximum) for the males and females.

Cranial feature	Present study		Sanborn (1949)	Simmons and Voss (1998)	
	Male	Female	Holotype	Male (n = 3)	Female (n = 2)
Greatest length of skull	20.0	21.0	20.5	19.54–20.39	19.71–20.49
Condylobasal length	18.0	18.5	18.5	18.06–18.72	17.99–19.07
Mastoid breadth	8.6	9.0	8.9	8.74–9.05	8.43–8.62
Zygomatic breadth	8.5	9.0	9.6	8.84–9.51	9.05–9.14
Breadth of braincase	7.0	7.5	8.2	7.92–8.22	7.97–8.26
Postorbital constriction	4	4	4.3	3.92–4.11	4.21
Palatal length	8	8	8.2	–	–
Length of upper tooth row	7	7	7.3	7.14–7.45	6.99–7.56
Length of lower tooth row	7	7	7.6	7.14–7.45	6.99–7.56
Length of mandible	13	13	–	–	–

## Discussion

Given the similarities with species of the genus *Carollia* Gray, 1838, the coloration of the pelage is not a diagnostic feature of the genus *Trinycteris*, although the two genera can be distinguished primarily by the absence of the central protuberances of the papilla in *Trinycteris* (Charles-Dominique et al. 2001, Rocha et al. 2013). *Trinycteris* can also be distinguished from the other Phyllostominae genera by the lack of papilla-like protuberances on the lips and chin, the tail enclosed by the interfemoral membrane, which does not extend to its posterior margin, and the lack of a layer of bare skin on the top of the head that joins the ears, in addition to craniometric measurements (Williams and Genoways 2008, Rocha et al. 2013, Reis et al. 2017). All these characteristics were observed in both specimens examined in the present study. The data on the COI gene revealed a high degree of similarity with the *T.nicefori* specimen from Costa Rica and a genetic divergence of less than 3% in comparison with the *T.nicefori* specimens from Guiana, which is consistent with the 3% DNA barcode threshold defined by Hebert et al. (2003). The genetic data also confirm the morphological identification and provided conclusive evidence of the occurrence of *T.nicefori* in Maranhão.

The body mass and craniometric parameters recorded in the present study were consistent with those reported by Sanborn (1949) and Simmons and Voss (1998), since the female presented body mass and craniometric measurements larger than in the male. In addition, morphological characters such as coat coloring and forearm measurements are consistent with descriptions provided by Sanborn (1949), Simmons and Voss (1998), Peracchi et al. (2011) and Reis et al. (2013, 2017).

The available data on *T.nicefori* show an important gap in the Amazon biome to the east of Brazil, indicating a disjunctive distribution between the Amazon and Cerrado biomes. The species is also absent from the Caatinga and Cerrado (*sensu stricto*) biomes, which indicates that it probably prefers mesic environments and is relatively intolerant to arid conditions (Rocha et al. 2013). In the present study, the distribution of *T.nicefori* was extended to the eastern extreme of the Amazon biome.

## Conclusion

The present study registered the occurrence of *T.nicefori* in the Brazilian state of Maranhão, based on the analysis of morphological and molecular data. This is the first record of the species for the state, where it was found in the Amazonian domain. This record extends the known distribution of *T.nicefori* 303 km to Godofredo Viana and 310 km to Cândido Mendes.
